# Effect of Age and Diabetes on the Response of Mesenchymal Progenitor Cells to Fibrin Matrices

**DOI:** 10.1155/2011/378034

**Published:** 2011-12-13

**Authors:** A. Stolzing, H. Colley, A. Scutt

**Affiliations:** ^1^Department of Cell Therapy, Fraunhofer Institute for Cell Therapy, Perlickstra*β*e 1, 04103 Leipzig, Germany; ^2^Academic Unit of Oral & Maxillofacial Medicine and Surgery, School of Clinical Dentistry, University of Sheffield, S10 2TA Sheffield, UK; ^3^Department of Engineering Materials and Sheffield Centre for Sports Medicine, University of Sheffield, S10 2RX Sheffield, UK

## Abstract

Mesenchymal stem cells are showing increasing promise in applications such as tissue engineering and cell therapy. MSC are low in number in bone marrow, and therefore *in vitro* expansion is often necessary. *In vivo*, stem cells often reside within a niche acting to protect the cells. These niches are composed of niche cells, stem cells, and extracellular matrix. When blood vessels are damaged, a fibrin clot forms as part of the wound healing response. The clot constitutes a form of stem cell niche as it appears to maintain the stem cell phenotype while supporting MSC proliferation and differentiation during healing. This is particularly appropriate as fibrin is increasingly being suggested as a scaffold meaning that fibrin-based tissue engineering may to some extent recapitulate wound healing. Here, we describe how fibrin modulates the clonogenic capacity of MSC derived from young/old human donors and normal/diabetic rats. Fibrin was prepared using different concentrations to modulate the stiffness of the substrate. MSC were expanded on these scaffolds and analysed. MSC showed an increased self-renewal on soft surfaces. Old and diabetic cells lost the ability to react to these signals and can no longer adapt to the changed environment.

## 1. Introduction

Adult tissue stem cells are thought to be involved in tissue maintenance and repair processes. When numbers are low, tissue stem cells are replenished by mesenchymal stem cells (MSC) via the blood system [1]. Depletion of the stem cell population has been suggested to contribute to a large number of degenerative diseases of brain, liver, skin, bone and plays a role in many aging-related diseases [2]. MSC reside in the bone marrow and can differentiate into a variety of tissues including bone, fat, and cartilage *in vitro* and *in vivo* [3, 4]. MSC are therefore ideal candidates for use in tissue engineering and cell therapy. MSC are particularly attractive because of the ease of isolation and manipulation.

It has been demonstrated that MSC *ex vivo* exhibit the characteristics typical of the Hayflick model of cellular senescence with a limited life span and in addition telomere shortening, accumulation of *β*-galactosidase [5], and impairment of differentiation [6]. Increased resistance to *in vitro *aging and extended expansion would be desirable characteristics for improved tissue engineering and cell therapy especially for the elderly. Several attempts to delay *in vitro* aging have been performed including reductions in oxygen level, temperature, glucose [7], genetic manipulation [8], and telomerase overexpression [9].

Many surfaces and scaffolds have been extensively evaluated for tissue engineering purposes. The effect of the mechanical stimulation of a particular surface on the behavior of MSC has been studied for a variety of potential differentiation effects. Mechanical stimulation either by vibrating cells, stretching cells or by providing surfaces with different mechanical properties can induce osteogenic differentiation or inhibit adipogenesis [10] through durable b-catenin activation [11].

Fibrin is a biodegradable polymer that is being increasingly used in tissue engineering applications and is showing promise as an alternative scaffold in vascular tissue engineering [12, 13] and skin [14]. Under physiological conditions, a fibrin clot is formed after trauma and the fibrin is responsible for most of the biological and mechanical properties of the blood clot [15]. The mechanical properties of fibrin clots are particularly important as they serve as both gap fillers to prevent bleeding and as a mechanical support to stabilise the wound. Because of this fibrin clots are remarkably extensible and elastic. The use of fibrin as a tissue engineering scaffold would therefore seem highly appropriate as in many ways the tissue engineering process could be considered to be a reiteration of the wound healing process.

Although a role in wound healing has been suggested for MSC, there is little direct biological evidence to support this. It has been suggested that fibrin can act as a form of “stem cell niche” for endothelial progenitor cells [10], and it would seem logical that this might also be the case with MSC. It is known that MSC can travel through the circulation and become incorporated into transplanted tissues [16–18] and fibrin has been shown to be highly haptotactic for a number of mesenchymal cell types including MSC [19, 20]. Research has been completed demonstrating that MSC are able to adhere, spread, and proliferate when seeded into a fibrin gel with low thrombin to fibrinogen ratios [21]. Stromal cells do not contract the fibrin and the material has no toxic effect on lapine MSC [11]. In addition fibrin can be isolated from the same donor as the MSC would therefore be a good material for clinical translation of cell preparations as the whole procedure would be performed using autologous material. However, there is lack of available data looking at the effects fibrin has on MSC growth and differentiation behaviour. We investigated the effect of fibrin on MSC from normal and diabetes type I rats as well as MSC from young and aged human donors. It is known that MSC from diabetic [22] and old donors [23, 24] do expand less *in vitro* and show earlier senescence. The aim was to establish a surface minimising *in vitro* aging and with good growth and differentiation potential. Growth and differentiation was evaluated on fibrin scaffolds with a range of stiffnesses to identify the optimal concentration of fibrin to support MSC.

## 2. Materials and Methods

### 2.1. Chemicals

All chemicals were obtained from Sigma-Aldrich (Dorset, UK) unless otherwise stated and used without further purification.

### 2.2. Cell Culture

Dulbecco's Modified Eagle Medium (Cambrex Bio Science, Workingham, UK) was supplemented with 10% Serum Supreme (Cambrex Bio Science, Workingham, UK), 1% Ultraglutamine (BioWhittaker, UK), and 1% penicillin-streptomycin solution and will hereafter be referred to as growth medium. For osteogenic differentiation cells were cultured in growth medium supplemented with dexamethasone (10^−8^ M) and ascorbate-2-phosphate (50 *μ*g/mL).

### 2.3. Mesenchymal Stem Cells

Wistar rats were purchased from Harlan (Bicester, UK) and kept on site according to home office regulations. To induce type I diabetes rats were injected with a single dose of streptozotocin (STZ 65 mg/kg body weight) in ice-cold 0.01 M sodium citrate buffer (pH 4.5) at 3-month old. Rats were allowed food and water *ad libitum*. Bodyweights were monitored weekly, along with urine glucose levels. Blood glucose levels were determined at the time of death. Rats of a similar age were used as healthy comparisons. Bone marrow cells were obtained centrifugally from tibiae and femurs according to the method of Dobson et al. [25] and MSC isolated by the method of Sekiya et al. [26].

Human MSC were purchased from Tulane University (Emeryville, Calif, USA) or AllCell (Emeryville, Calif, USA). MSC isolated from donors aged between 18–25 were classified as young whilst MSC isolated from donors aged 55–60 were classified as old.

### 2.4. Immunophenotyping of Mesenchymal Stem Cells

To confirm MSC phenotype, human MSC were harvested after 7 days and stained with antibodies against CD11, CD31, CD44, CD45, CD90, CD105, D7fib, and stro-1 (diluted 1 : 100; 4°C; 30 min; Serotec, Oxford, UK). Rat MSC were stained for CD11, CD44, CD45, CD90, and CD105. Cell phenotyping and cell size were performed using a personal flow cytometry system (GUAVA Instruments, Hayward, USA, or an Acuri FACS).

### 2.5. Demonstration of Differentiation Potential of Mesenchymal Stem Cells

20,000 cells were seeded in 24-well plates and incubated in osteogenic medium (containing 10^−8^ M dexamethasone and 50 *μ*g/mL ascorbate-2-phosphate for 14 days. MSC were fixed and stained histochemically for alkaline phosphatase activity. Briefly, MSC were incubated for 30 min at room temperature in a solution of naphthol AS-BI phosphate (0.05 mg/mL) in Tris buffer (0.08 M, pH 7.5) containing fast red bb or fast violet (1 mg/mL), washed, and photographed. For adipogenic differentiation 20,000 cells were seeded in 24-well plates and incubated in adipogenic differentiation medium (1 mM dexamethasone, 5 mg/mL insulin, 0.5 mM isobutylmethylxanthine (IBMX), 60 mM indomethacin) for 14 days. Cells were fixed with isopropanol and stained with oil red O solution to detect the presence of neutral lipid vacuoles and photographed.

### 2.6. Production of the Fibrin Scaffolds

Fibrinogen type I from bovine plasma was dissolved in medium at 37°C for a range of concentrations (3, 10, and 30 mg/mL). The mixture was gently agitated until the fibrinogen dissolved and then cooled by refrigeration. Thrombin from bovine plasma was added (1 unit/mL) to induce gelation. 5 mL was added to each 25 cm^2^ culture flask. Flasks were incubated at 37°C for 1 h and rinsed with PBS before use. The MSC were seeded on top of the fibrin scaffold.

### 2.7. Colony-Forming Unit Fibroblastic Assay

To determine proliferation capacity and maintenance of differentiation potential the colony-forming unit fibroblast assay was employed as a modification of the technique originally described by Kuznetsov et al. [27]. Primary MSC were cultured on fibrin gels (3, 10 and 30 mg/mL) and tissue culture plastic (TCP) controls for 7 days. 1000 cells were then transferred to 55-cm^2^ plates and cultured for a subsequent 14 days in osteogenic medium. At this point colonies were fixed in ethanol, sequentially stained for alkaline phosphatase and total colonies and photographed as previously described [28].

### 2.8. Colony Analysis

Colony numbers were assessed using the method of Dobson et al. [25]. Briefly, the acquired digital images were imported into Photoshop, colony irregularities smoothed out and converted into a 256-level greyscale format. The greyscale images were then imported into Bio-image “Intelligent Quantifier” and the colony number calculated.

### 2.9. Data Analysis

Values were expressed as mean ± standard deviation of three experiments. Comparison of results was performed by one-way analysis of variance (ANOVA) and multiple comparisons made using Tukey's test. Data was considered significantly different when *P* < 0.05.

## 3. Results

### 3.1. Clonogenic and Osteogenic Differentiation Potential of Healthy and Diabetic Rat Mesenchymal Stem Cells after Preculture on Fibrin

**Figure 1:**
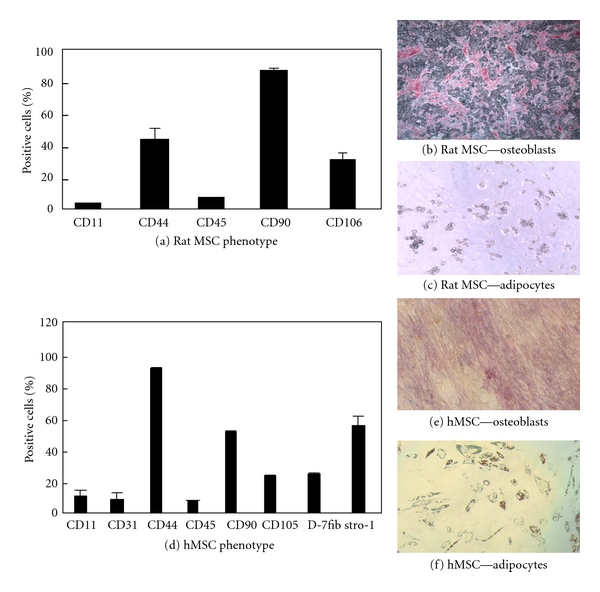
MSC validation. MSC isolated from humans and rats were immunophenotyped using typical MSC marker (Figures 1(a) and 1(b)), and the stem cell potential was confirmed by differentiating them into adipocytes and osteoblasts (Figures 1(c) and 1(d)).

MSC were isolated from normal or streptozotocin type I diabetic rats (2-3-month old) and their phenotype confirmed by flow cytometric analysis. Cells were CD44 and CD90 positive, CD45 low, and negative for CD11 (Figure 1(a)). The cells were able to differentiate into adipocytes and osteoblasts under appropriate culture conditions (Figure 1(b) and 1(c)).

To determine the effects on the clonogenicity and retention of differentiation potential of culturing MSC on three-dimensional fibrin scaffolds with physiologically relevant elastic moduli, freshly isolated MSC were cultured on 3, 10 or 30 mg/mL fibrin and on TCP for 7 days before transferring 1000 cells to uncoated Petri dishes and culturing for a further 14 days in osteogenic growth medium. We found that MSC derived from young rats showed increased CFU-f frequencies compared to MSC derived from rats 12 weeks after streptozotocin injection.

**Figure 2:**
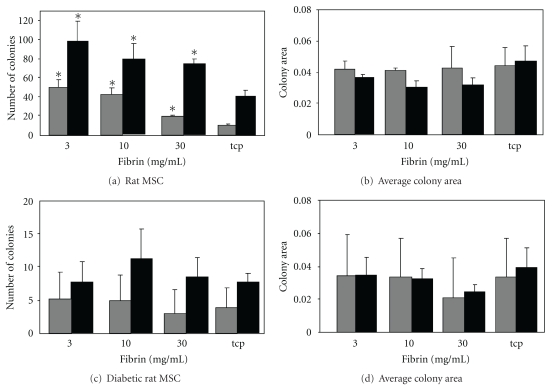
Effects of fibrin surfaces on healthy and diabetic rat MSC. MSC isolated from young and diabetic rats were grown on fibrin gels for 7 days, and then 1000 cells were seeded into the CFU assays under osteogenic conditions. ALP-positive (grey) and total colony numbers (black) were calculated for normal rat MSC (a) as well as the mean size of the colonies (b). CFU numbers for MSC from were also calculated (c) as well as the mean colony size (d). *Denotes a statistically significant difference from the other groups.

Frequency of ALP colonies was significantly increasing on fibrin in comparison to TCP (Figure 2(a)) but the average colony size was not affected (Figure 2(b)). The same was observed for the frequency of total colonies (Figure 2(c)) and the average colony size of these (Figure 2(d)). The initially lower colony numbers from diabetic rats was not modulated by culturing on fibrin and the same trend was found with regards to the CFU size (total colony count) and the number and size of the ALP-positive colonies (Figures 2(b) and 2(d)).

### 3.2. Clonogenic and Osteogenic Differentiation Potential between Old and Young Human Mesenchymal Stem Cells

Young MSC were isolated from 18–25-years-old donors and old MSC from donors aged between 55 and 60 years. Cell phenotype was confirmed by flow cytometric analysis. Cells were CD44, CD90, stro-1, and D7fib positive and CD11, CD31, and CD45 low (Figure 1(d)). The cells were able to differentiate into osteoblasts and adipocytes under the appropriate culture conditions (Figures 1(e) and 1(f)).

**Figure 3:**
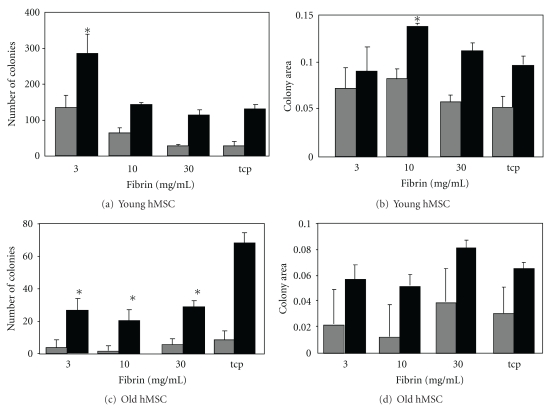
Effects of fibrin surfaces on young and old human MSC. Isolated MSC from young donors and aged donors were grown on fibrin gels for 7 days, and then 1000 cells were seeded into the CFU assays under osteogenic conditions. The number of ALP-positive colonies (grey) and total colony numbers (black) were calculated for young (a) and aged (c) MSC. Mean sizes of colonies were calculated for young (b) and aged (d) donor MSC. *Denotes a statistically significant difference from the other groups.

The number of colonies of expanded young human MSC was positively linked to the softness of the fibrin substrate. MSC were cultured on the different surfaces and then reseeded onto TCP for the CFU-f assay. The number of ALP-positive colonies increased significantly after culture on 3 and 10 mg/mL fibrin relative to TCP (Figure 3(a)). Total colony numbers followed the same trend; however only the 3 mg/mL fibrin leads to a significant increase in colony numbers. The size of colonies was slightly but not significantly increased when expanded on 10 mg/mL fibrin gels (Figure 3(b)).

### 3.3. Response of Old MSC Cultured on Fibrin

**Figure 4:**
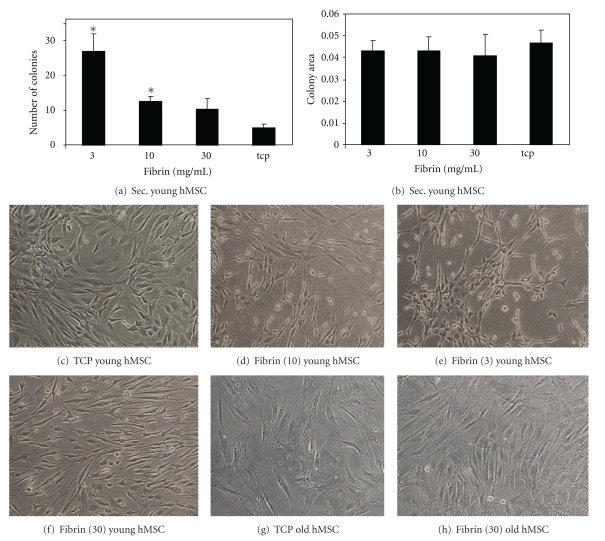
MSC morphology and self-renewal. MSC from an 18-year-old healthy donor were cultured on 10 mg/mL fibrin for 7 days before re-seeding them onto different concentrations of fibrin or TCP. We call these secondary colonies. The different surfaces were again tissue culture plastic, 3 mg/mL fibrin gel, 10 mg/mL, and 30 mg/mL (d). MSC from a 55-year-old donor are cultured on TCP and 30 mg/mL fibrin gel. Colony numbers (a) as well as size (b) are shown and the different morphologies (c–h) are shown. *Denotes a statistically significant difference from the other groups.

In general the number of CFU derived from cultured MSC from old donors is much lower compared to the MSC from young donors (Figure 3). Interestingly, we see the opposite trend for CFU numbers using old MSC expanded on the different surfaces compared to young MSC. We find significantly more colonies on TCP and less on the softer surfaces, with no difference between fibrin concentrations. The trend is not significant for ALP-positive colonies but for total colonies. For MSC from aged donors we find *∼*50% ALP-positive colonies compared to MSC from young donors. Total colony numbers are decreased by *∼*40%. If we compare this to the fibrin concentration which gave the highest CFU numbers with the MSC from young donors we find a much greater decrease. On 3 mg/mL fibrin we only find 5% ALP-positive colonies and *∼*10% total colonies using MSC from aged donor. Colony sizes were also slightly larger on plastic and the 30 mg/mL fibrin but did not reach significance (Figures 3(b) and 3(d)). Also the old MSC do become smaller on the fibrin surfaces on all tested concentrations (Figures 4(c)–4(h)). On the plastic they have an enlarged morphology typical for senescent MSC, and on the highest concentrated fibrin gel we find them to be much smaller and highly aligned (Figures 4(f) and 4(h)). MSC on the other two concentrations followed the same pattern as MSC from young donors (data not shown).

### 3.4. Effect of Fibrin on Expansion and Differentiation of Secondary Mesenchymal Stem Cells

To assess whether the effect of substrate stiffness on the clonogenic potential of MSC was still present after an initial expansion on the 10 mg/mL fibrin preparation (showing the highest CFU-f numbers), MSC were re-seeded onto the different fibrin substrates (3–30 mg/mL) to see if the cells retained the influence by the first substratum. Secondary MSC followed the same trend as the primary MSC. An increase in the number of colonies per dish after preculture on the fibrin compared to the TCP control was observed; however this time also the 10 mg/mL fibrin-cultured MSC showed a significant difference in comparison to TCP-cultured MSC (Figure 4(a)). However, unlike the primary human MSC, no difference in colony size was seen between the different groups (Figure 4(b)).

### 3.5. Morphological Comparison

MSC cultured on fibrin changed their morphology according to the fibrin softness (Figures 4(c)–4(h)). The cell size was reduced on all fibrin surfaces. This was confirmed by analysing cell size by flow cytometry (Table 1). On the 30 mg/mL fibrin, and to a lesser extent on the 10 mg/mL fibrin, the MSC were highly aligned. Furthermore, on the 3 mg/mL surfaces the cells started to form networks. As this hinted towards a potential better self-renewal we cultured these MSC for one passage on the different fibrin concentrations and analysed second-generation colony formation. The MSC cultured on plastic lost about 90% of their colony-forming capacity. The frequency of CFU could be increased by a factor of about 5 when the cells were cultured on 3 mg/mL fibrin; however no effect on proliferation rates was seen (Figures 4(a) and 4(b)).

### 3.6. Effect of Fibrin Concentration on Gel Stiffness

**Figure 5:**
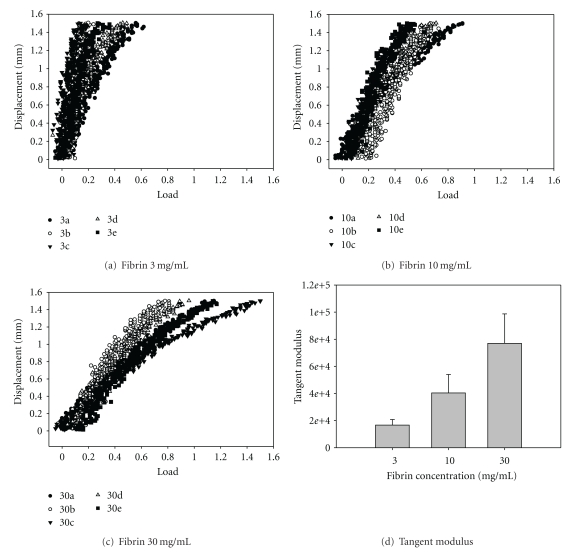
Tangent modulus of a range of fibrin gel produced with varying fibrinogen concentrations. Fibrin gels were made by mixing fibrinogen (3, 10, and 30 mg/mL) with thrombin (1 *μ*l/mL). Tensile testing of the gels was performed using a Bose ElectroForce 3200 (a–c). The tangent moduli were calculated from the slope of the plot of stress versus strain at 20% strain (*n* = 6) (d).

One possible factor contributing to the effects of fibrin concentration on MSC clonogenic capacity is its effects on fibrin gel stiffness. To determine the effect of fibrin on gel stiffness a stress strain plot was calculated using a Bose Electroforce 3200 (Figures 5(a)–5(c)). As no linear region was found, the stiffness was measured using a tangent modulus at 20% strain. The data showed that as the concentration of fibrinogen increased so did the stiffness with a modulus of 16.7 kPa for 3 mg/mL, 40.4 kPa for 10 mg/mL, and 77 kPa mg/mL for 30 mg/mL. The stiffness of the 30 mg/mL gel was statistically difference from the 3 and 10 mg/mL gel (Figure 5(d)).

## 4. Discussion

MSC are becoming increasingly popular for tissue engineering and cell-based therapies due to the ease of isolation and cultivation. Their immunomodulatory functions as well as their secretome make them an ideal source for therapies. However *in vitro* expansion is needed to expand numbers, as MSC occur in low numbers (1 of 100,000 cells) in bone marrow [29] and decline in frequency with age [24]. Expansion leads to *in vitro* aging of the MSC accompanied with a declining proliferation and differentiation potential [30]. Aged MSC express markers typical of aged somatic cells including increased p53 and p21 levels [31], have a changed morphology [3], and express the senescence marker *β*-galactosidase [23]. Clinical use of aged MSC could lead to serious side effects as it is known that senescent cells can induce cancer formation [32]. Human adipose-tissue-derived mesenchymal stem cells can spontaneously transform when they reach senescence [33]. Because of this, it is necessary to keep the numbers of senescent cells present in tissue-engineered constructs as low as possible and to minimize senescence during expansion. It is now well acknowledged that stem cells interact with their environment [34]. Manipulation of stem cell behaviour by changing the niche of the stem cells is possible. Extracellular matrix components are part of the stem cells niche. Fibrin is a natural biodegradable polymer increasingly used in tissue engineering and can be produced autologously and is therefore a good choice of scaffold for potential therapeutic applications. It is widely used in cartilage tissue engineering [35] and can be rapidly degraded by plasmin. It is therefore an ideal candidate for *in vitro* expansion of MSC and could also be used for transplantation to protect and modulate MSC behaviour.

Fibrin gels with various fibrinogen concentrations were made with different mechanical properties (different Young's moduli). MSC were seeded onto these gels and cultured before being analysed. We found that MSC from young rats and MSC from young humans responded to the different fibrinogen concentrations. The highest self-renewal measured using the CFU-f assay capacity was found on the softest fibrin surfaces. This effect however was lost for MSC from either diabetic Type I rats or aged human donors. We know from former studies that CFU-f numbers from diabetic rats and aged humans [24] were lower compared to young healthy controls. This is now confirmed for the frequency of culture-expanded MSC with the same trends observed. The use of the CFU-f as an assay to determine the number of MSC present in a cell preparation has been well characterised [25], with the assumption that each colony arises from a single cell allowing for the number of colonies to be correlated to the number of stem cells in the original sample. Fibrin gels seem to alter the frequency of stem cells in the MSC cultures and can increase in young MSC the number of MSC with an high proliferative and differentiation potential. MSC cultured on fibrin appeared to align along the same axis whereas on TCP they had a typical “cobble-stone-” like appearance. Not only were differences seen in the morphology of the cells but also the size of the cell was different. It is well accepted that an increase in cell size is linked to senescence of the cell and thereby suggests that smaller cells are “younger” than the larger ones [23]. The MSC from old donors decreased in size after culturing on the soft fibrin surface, maybe due to matrix-induced rejuvenation effects. In fibroblasts the senescence phenotype could be reversed by altering cell attachment via the inhibition of calveolin-1 [36].

It was observed that not only CFU-f efficiency, but also the number of colonies that stained positive for ALP was higher after pre-culture on the softer substrates for cells from young and nondiseased donors. This data suggests that pre-culturing on the softer substrates maintains a more “stem-cell-” like phenotype. A study investigating maintenance of human MSC phenotype has implicated the basement membrane ECM as a critical factor in the proliferative life span and maintenance of differential potential of stem cells in culture and highlights the importance of a native, soft substratum for maintaining a more “stem cell-” like phenotype and showed greater effects than growth factors like fibroblastic growth factor [37]. Studies on the effect of temperature on MSC from old donors showed the same reduced response to an environmental challenge [38]. Proteomic studies on human aged MSC showed that antioxidant and cytoskeleton functions are diminished in aged MSC [39]. The beneficial effects of surfaces on the “stemness” properties could be mediated via the cytoskeleton, or due to changes in the signal transduction pathway. Cell shape was shown to regulate stem cell differentiation [40]. The Rho family of small GTPases transmit signals from the extracellular matrix to the actin skeleton and the nucleus, modulating gene expression [36, 41]. Receptors involved in the signal transmission are integrins, dystroglycans, syndecans, CD44, and Rhamm. Such signals can lead to dramatic changes in cell morphology and cell architecture involving cytoskeleton and chromatin structures, affecting gene expression [42]. Lamins making up the nuclear envelope are connected to the actin filaments through nespin, anchoring the nucleus to the cytoskeleton. Disruption of actin leads to global histone deacetylation and cells rounding up [43].

**Outlook**

This is the first report of the self-renewal potential of expanded MSC from aged and diseased donors on fibrin scaffolds. We have shown that young MSC maintain a better self-renewal on soft fibrin gel, but this benefit is partly lost when cells from aged or diabetic organisms are used. Additional modifications of the scaffolds might be necessary to gain the same effects seen in young MSC.

## Figures and Tables

**Table 1:** Cell size grown on fibrin. Fibrin gels were made by mixing fibrinogen (3, 10, and 30 mg/mL) with thrombin (1 *μ*l/mL). Different MSC types were analysed for cell sizes.

Cell type	Abitary units
Young rMSC	329 ± 136
Diabetic rMSC	392 ± 150
Young rMSC (30 mg/mL)	420 ± 10
Young hMSC	408 ± 51
Aged hMSC	420 ± 57
Aged hMSC (30 mg/mL)	344 ± 127
